# Prevalence and 10-Year Stability of Personality Disorders From Adolescence to Young Adulthood in a High-Risk Sample

**DOI:** 10.3389/fpsyt.2022.840678

**Published:** 2022-03-24

**Authors:** Delfine d’Huart, Martin Steppan, Süheyla Seker, David Bürgin, Cyril Boonmann, Marc Birkhölzer, Nils Jenkel, Jörg M. Fegert, Marc Schmid, Klaus Schmeck

**Affiliations:** ^1^Department of Child and Adolescent Psychiatric Research, University Psychiatric Clinics Basel, Basel, Switzerland; ^2^Division of Developmental and Personality Psychology, University of Basel, Basel, Switzerland; ^3^Department of Child and Adolescent Psychiatry and Psychotherapy, Ulm University, Ulm, Germany; ^4^Department of Forensic Child and Adolescent Psychiatry, University Psychiatric Clinics Basel, Basel, Switzerland

**Keywords:** personality disorders **(PDs)**, prevalence, stability, high-risk sample, youth

## Abstract

**Background:**

With the implementation of the 11th edition of the International Classification of Diseases (ICD-11) in early 2022, there will be a radical change in the framework and process for diagnosing personality disorders (PDs), indicating a transition from the categorical to the dimensional model. Despite increasing evidence that PDs are not as stable as previously assumed, the long-term stability of PDs remains under major debate. The aim of the current paper was to investigate the categorical and dimensional mean-level and rank-order stability of PDs from adolescence into young adulthood in a high-risk sample.

**Methods:**

In total, 115 young adults with a history of residential child welfare and juvenile-justice placements in Switzerland were included in the current study. PDs were assessed at baseline and at a 10-year follow-up. On a categorical level, mean-level stability was assessed through the proportion of enduring cases from baseline to follow-up. Rank-order stability was assessed through Cohen’s κ and tetrachoric correlation coefficients. On a dimensional level, the magnitude of change between the PD trait scores at baseline and at follow-up was measured by Cohen’s *d*. Rank-order stability was assessed through Spearman’s ρ.

**Results:**

The prevalence rate for any PD was 20.0% at baseline and 30.4% at follow-up. The most frequently diagnosed disorders were antisocial, borderline, and obsessive-compulsive PDs, both at baseline and at follow-up. On a categorical level, the mean-level stability of any PD was only moderate, and the mean-level stability of specific PDs was low, except of schizoid PD. Likewise, the rank-order stability of any PD category was moderate, while ranging from low to high for individual PD diagnoses. On a dimensional level, scores increased significantly for most PDs, except for histrionic traits, which decreased significantly from baseline to follow-up. Effect sizes were generally low. The rank-order stability for dimensional scores ranged from low to moderate.

**Conclusion:**

The findings indicate low to moderate stability of Pds and Pd traits from adolescence to adulthood, which supports the growing evidence that categorical diagnoses of Pds are quite unstable. This in turn, emphasizes the use of the upcoming ICD-11 that Acknowledgments Pds to be only “relatively” stable.

## Introduction

The introduction of personality disorders (PDs) in the third edition of the Diagnostic and Statistical Manual of Mental Disorders (DSM-III) (1) led to a substantial increase in empirical research and clinical interest (2). Yet, the advent of specific diagnostic criteria and a multiaxial approach that differentiated PDs (i.e., Axis II) from clinical syndromes (i.e., Axis I) set the stage for an ongoing controversy about the conceptualization and diagnosis of PDs. While PDs were defined as discrete, distinct categories, the shortcomings of such a categorical classification model became quickly apparent (3–5), and a shift to a more dimensional model, in which PDs are perceived as extreme variants of normal personality dimensions, became inevitable (6, 7). With the upcoming 11th edition of the International Classification of Diseases (ICD-11) (8), the conceptualization of PDs is finally in transition, acknowledging PDs to be only “relatively” stable (9–11). For over decades, however, temporal stability consisted in one of the major distinguishing features between Axis I and Axis II disorders with the stability of PDs being substantially higher than for other mental disorders. Yet cumulative findings slowly appeared to question the stability of PDs, by suggesting considerable improvement over time (12, 13). Thus, against the common assumption that PDs are “enduring,” “inflexible,” and “stable” the categorical stability of PDs has found to be not much higher than the stability of other mental disorders (14). Indeed, the Collaborative Longitudinal Study of PDs (CLPS) (15), which investigated the stability of schizotypal, borderline, avoidant, and obsessive-compulsive PDs over time, found that fewer than half of PD patients still met the criteria for a diagnosis after 2 years (16). With regard to borderline PD (BPD), 85% of the original sample had remitted after 10 years (17).

Nevertheless, as outlined in Morey and Hopwood’s narrative review (18), temporal stability is a complex notion and has to be examined with respect to several factors. First, estimates tend to vary as a function of the type of stability being assessed. In the present study, the focus relies on the two types of stability that have been studied most frequently, namely mean-level and rank-order stability. Mean-level stability refers to the degree to which the average level of a PD or a PD trait in a given sample changes over time. Rank-order stability, on the other hand, refers to the consistency of an individual’s relative ordering compared to others in a given sample, capturing, thus, the extent to which interindividual differences persist over time (18). Rank-order stability is high if the participants in a given sample maintain their ordering with regard to a specific PD or PD trait relative to each other over time, even if the sample as a whole increases or decreases with regard to that PD or PD trait. As such, rank-order changes are independent of mean-level changes (19). Second, estimates depend in part on the type of PD construct being assessed (i.e., categories or traits), suggesting higher stability for dimensional traits rather than for distinct categories (20–22). In their narrative review, Grilo and McGlashan (21) reported that the rank-order stability for meeting any PD diagnosis is fair to moderate, while individual PD diagnoses often exhibit lower stability. In contrast, dimensional scores tend to show slightly higher stability estimates. Durbin and Klein (20) confirmed these findings by showing that rank-order stability was low to fair for categorical PD diagnoses over a 10-year follow-up in depressed outpatients, while rank-order stability for dimensional PD traits was fair to moderate. According to Grilo et al. (23), mean-level stability, when assessed dimensionally, is generally lower than rank-order stability, which indicates that symptoms tend to decrease on average, but the rank-ordering of individuals within a defined sample remains roughly the same. Third, estimates may be affected by the assessment method being used to measure PDs. Self-report questionnaires tend to show a relatively higher stability than clinical interviews (20, 24). For instance, the findings from Samuel et al. (22) for dimensional ratings showed significantly greater rank-order and mean-level stability for self-report questionnaires compared to clinical interviews. Findings regarding categorical PD diagnoses, in contrast, indicated comparable rank-order and mean-level stability. Finally, Morey and Hopwood (18) outlined how the clinical status and age range of a given sample are critical factors affecting PD stability estimates over time. Studies investigating the course of PDs, however, seem to focus mainly on adult samples, and studies on children and adolescents are scarce. This paucity of research has been in part due to the widespread reluctance to diagnose PDs in youth (25, 26) and to the belief that personality in adolescence is inconstant and characterized by emotional outbursts and impulsive behavior (27, 28). Existing literature, however, clearly states that PDs can be validly and reliably diagnosed among juveniles (27, 28) and that the stability of PDs in adolescence is found to be comparable to the stability in adulthood (29, 30).

Given the apparent number of developmental tasks [e.g., achieving emotional independence from parents, developing close relationships with peers, preparing for a professional occupation (31)], the transition from adolescence to adulthood seems to be a salient period for investigating the stability of PDs (18, 32). To the best of our knowledge, however, only two studies have explicitly investigated the stability of PDs from adolescence to early adulthood. The Children in the Community (CIC) study investigated the stability of PD traits in a community sample ranging in age from 9 to 28 (33). Findings show that mean PD traits were highest in adolescence and declined linearly to adulthood, although effect sizes were small. Rank-order stability was found to be low to moderate, and cluster C traits seemed to be less stable than cluster A and B traits (34). Similarly, Bornovalova et al. (35), who investigated the stability and heritability of BPD in a community sample, showed a significant mean-level decline from age 14 to 24, although rank-order stability was high. A third study, namely the study from Chanen et al. (36), investigated the 2-year stability of PDs in older adolescent outpatients, aged 15–18 years, and found that 74% of those diagnosed with a PD at baseline still met the criteria for a PD at follow-up. Regarding dimensional ratings, both rank-order and mean-level stability ranged from low (PD NOS) to moderate (borderline, histrionic, and schizotypal) to high (antisocial and schizoid) (36).

Given the apparent role of developmental influences on the etiology of PDs, studies about the stability of PDs in high-risk samples are surprisingly lacking. The aim of the present study was therefore to examine the prevalence of PDs and their stability over a 10-year period from adolescence to adulthood in adolescents placed in residential care and juvenile-justice institutions. Due to multiple risk factors – such as childhood adversities (37), unfavorable parenting practices, low socioeconomic status, parental mental disorders (38), early mental-health problems (e.g., ADHD, oppositional defiant disorders, and attachment disorders), symptoms of depression and anxiety (39), substance use (40), self-harming behavior (41), psychopathic traits, and youth delinquency (42) – adolescents in residential care and juvenile-justice institutions are particularly at risk of developing a PD, and PD prevalence rates among them are high, ranging from 18 to 40% across studies (43–45). To account for conceptual and methodological factors, both categorical and dimensional mean-level and rank-order stability were investigated.

## Materials and Methods

### Study Design

#### Baseline

Data was obtained from the longitudinal “Swiss Study for Clarification and Goal-Attainment in Child Welfare and Juvenile-Justice Institutions” [German: Modellversuch zur Abklärung und Zielerreichung in stationären Massnahmen (MAZ)] (46). The study was conducted between 2007 and 2011 with the primary aims of describing the mental health of children and adolescents in residential care and of investigating the effects of residential youth care over an approximately 1-year period in Switzerland. Child welfare and juvenile-justice institutions accredited by the Swiss Federal Ministry of Justice were invited to participate, of which 64 institutions agreed to take part. Juveniles who had been living for at least 1 month in 1 of these 64 included child welfare and juvenile justice institutions and possessed sufficient language skills in German, French, or Italian as well as sufficient intelligence scores (IQ > 70) were eligible for participation. The juveniles had been placed in the child welfare and juvenile-justice institutions by penal law, by civil law, or voluntarily. Both voluntary placement and placement by civil law were due to severe mental distress or precarious living conditions. Prior to participation, juveniles, parents or legal guardians, and social workers were asked to provide informed consent. Participants then completed computer-administered questionnaires as well as semistructured clinical interviews regarding mental health, psychosocial problems, and offending behavior. Assessment was conducted by trained psychologists and research assistants. Overall, 592 children and adolescents aged 6–26 years (mean age = 16.3 years) participated at baseline. Of those participants, 511 agreed to be contacted for a possible follow-up study. The study procedure was approved by the Ethics Committees on Research Involving Humans at the University of Basel and the University of Lausanne (Switzerland) and by the Institutional Review Board at the Ulm University (Germany).

#### Follow-Up

After a follow-up period of approximately 10 years, participants were reassessed in the study “Youth Welfare Trajectories: Learning from Experiences” [German: Jugendhilfeverläufe: Aus Erfahrung Lernen (JAEL)], which is currently being conducted to examine participants’ psychosocial development over time and their transition out of care. Participants were contacted by postal mail, phone, email, and social media. Of the 511 participants, 231 (45.2%) agreed to participate in the follow-up. Despite considerable efforts, 8 (1.6%) participants could not be located, 121 (23.7%) could not be reached, 99 (19.4%) refused to participate, 44 (8.6%) did not provide informed consent, and 8 (1.6%) were deceased. A study flow-chart is provided in Supplementary Figure 1. An analysis of the sample attrition showed no significant differences in sociodemographic features (i.e., age, gender, number of former placements, and average duration in residential care) between the participants who took part in the follow-up and those who did not. The follow-up assessment consisted primarily of a set of online questionnaires that participants could complete from home. Participants were then invited to a face-to-face meeting, where they were reassessed using semistructured clinical interviews and semistructured qualitative in-depth interviews regarding mental health, psychosocial problems, and offending behavior. Assessment was conducted by trained psychologists, doctoral students, and research assistants. The study procedure was approved by the Ethics Committee Northwestern and Central Switzerland (EKNZ, Ref.: 2017-00718).

### Participants

As the primary aim of this study was to investigate the stability of PDs from adolescence to adulthood, only participants with complete data from the Structured Clinical Interview for DSM-IV-TR Axis II Personality Disorders (SCID-II) (47) at baseline and at follow-up were included, which left a study sample of 138 participants. In addition, participants younger than 12 years of age or older than 18 years at baseline were excluded. The final sample included 115 participants (39.13% female) with a mean age of 15.82 (*SD* = 1.93; range 12–18) at baseline and a mean age of 25.89 (*SD* = 2.18; range = 21–30) at follow-up (Table 1). Excluded participants revealed no statistically significant differences from participants at baseline in age [*t*(169) = -1.54; *p* = 0.126], gender [χ^2^(1) = 0.002; *p* = 0.964], number of placements in residential care [*t*(551) = 0.40; *p* = 0.689], average duration in residential care [*t*(228) = -0.19; *p* = 0.849], PDs [χ^2^(1) = 2.41; *p* = 0.120], and mental-health problems other than PDs [χ^2^(1) = 0.56; *p* = 0.451].

**TABLE 1:** Sample characteristics at baseline and follow-up (*N* = 115).

	Baseline	Follow-up
	
	*M (SD)*	*M (SD)*
Age (years)	15.8 (1.9)	25.9 (2.2)
Number of placements in residential care	0.7 (1.0)	3.4 (2.8)
Average duration in residential care (years)	1.4 (1.7)	6.3 (4.8)
	*n* (%)	*n* (%)
Gender (female)	45 (39.1)	45 (39.1)
**Current mental-health disorders^a^**		
Any current mental-health disorder	74 (64.9)	64 (55.6)
ADHD^b^	13 (11.4)	24 (20.9)
Anxiety disorder^b^	29 (25.4)	19 (16.5)
Conduct disorder^b,c^	34 (29.8)	
Mood disorder^b^	16 (14.0)	22 (19.1)
Personality disorder	23 (20.0)	35 (30.4)
Psychotic disorder^b^	2 (1.7)	2 (1.7)
PTSD^b^	5 (4.4)	6 (5.2)
Substance-use disorder^b^	17 (14.9)	41 (35.6)
Current mental-health treatment^d^	55 (61.1)	27 (23.5)

*^a^Participants with multiple mental-health disorders are displayed more than once.*

*^b^Due to missing data, the sample size at baseline was N = 114. ^c^Only available at baseline. ^d^Due to missing data, the sample size at baseline was N = 90.*

### Measurements

#### Sociodemographic Characteristics

Sociodemographic information – age, gender, number of former placements, average duration in residential care (i.e., total time spent in residential care and juvenile-justice institutions), and current mental-health treatment – was collected using a computer-based questionnaire at baseline and at follow-up. Participants’ data on social welfare, disability, and unemployment insurance were only assessed at follow-up.

#### Mental Disorders

Mental disorders at baseline were assessed with the Schedule for Affective Disorders and Schizophrenia for School-Age Children – Present and Lifetime Version (K-SADS-PL) (48). The K-SADS-PL is a semistructured clinical interview that provides a reliable and valid measurement of DSM-IV diagnoses in children and adolescents. At follow-up, mental disorders were examined with the Structured Clinical Interview for DSM-5 Disorders – Clinician Version (SCID-5-CV) (49). The SCID-5-CV is a semistructured clinical interview based on DSM-5 diagnoses covering the most common diagnoses seen in clinical settings: depressive and bipolar disorders, schizophrenia spectrum and other psychotic disorders, substance-use disorders, anxiety disorders, obsessive-compulsive disorder, post-traumatic stress disorder (PTSD), attention-deficit hyperactivity disorder (ADHD), and adjustment disorder. In addition, the SCID-5-CV screens for 17 additional DSM-5 diagnoses. Items and diagnoses are scored based on dichotomous “present” and “absent” response options. The SCID-5-CV presents excellent reliability, with Cohen’s κ ranging from 0.70 to 0.75 (50).

#### Personality Disorders

Personality disorders were assessed at baseline and at follow-up using the SCID-II (47). The SCID-II is a semistructured interview designed to yield PD diagnoses based on the DSM-IV and DSM-IV-TR (i.e., paranoid, schizoid, schizotypal, histrionic, borderline, antisocial, narcissistic, avoidant, dependent, obsessive-compulsive, depressive, and passive-aggressive PDs) and consists of 134 items, which are rated on a 3-point Likert scale (1 = absent, 2 = subthreshold, and 3 = threshold). Since depressive and passive-aggressive PDs were removed in the DSM-5, both disorders were included in the PD NOS section in the following analyses. Categorical diagnoses are provided according to the specific diagnostic thresholds of PDs the DSM-IV. Dimensional scores are provided by summing the scores from each individual item for each separate PD. Interrater reliability for categorical diagnoses varies from 0.48 to 0.98 (Cohen’s κ), and internal consistency ranges from 0.71 to 0.94 (51). At baseline, the diagnosis of antisocial PD was assigned only if study participants were over 18 years old. Due to participants’ young age, most of them could not be given the diagnosis. To anticipate later analyses of the stability of antisocial PD, the criteria for antisocial PD were nevertheless collected for participants both under and over 18 years old. The present analyses therefore include antisocial PD diagnoses in participants who were both younger and older than 18 years old at baseline.

### Statistical Analysis

First, to determine the prevalence rates of PDs at baseline and at follow-up, we performed descriptive statistical analyses. Group comparisons regarding social benefits between participants with and without a PD were assessed at follow-up using χ^2^ tests. Second, categorical mean-level stability was measured by the proportion of enduring cases from baseline (t1) to follow-up (t2), that is, the number of participants meeting the criteria for a PD at both measurement times divided by the total number of participants with a PD at baseline. Categorical rank-order stability was calculated by Cohen’s κ and tetrachoric correlations (*r*_*tet*_). Cohen’s κ is one of the most commonly used statistics to test diagnostic agreement between diagnoses assigned at baseline and at follow-up. A negative value indicates an agreement worse than expected or even a disagreement. A value between 0 and 0.20 represents a low agreement, and a value ranging from 0.21 to 0.40 a fair agreement. A κ between 0.41 and 0.60 indicates a moderate agreement, a κ between 0.61 and 0.80 a substantial agreement, and 0.81–1.0 a perfect agreement between two assessments (52). While Cohen’s κ takes into account the possibility of an agreement occurring by chance, tetrachoric correlation coefficient (*r*_*tet*_) measures the mere relationship between binary baseline and follow-up scores with the assumption of bivariate normality (53). Similar to Pearson’s *r*, a value between 0.1 and 0.3 is considered to be low, a value between 0.3 and 0.5 moderate, and a value between 0.5 and 0.8 high. Finally, for dimensional PD ratings, mean-level stability was measured by calculating mean trait scores and standard deviation at baseline and at follow-up, resulting in a mean-difference score. Cohen’s *d* was used to estimate the effect size of the magnitude of change between baseline and follow-up scores. According to Cohen (54), an effect size of 0.20 is considered a small effect, an effect size of 0.50 a moderate effect, and an effect size of 0.80 a large effect. Dimensional rank-order stability was measured using Spearman’s ρ (*r*_*s*_), given a substantial positive skew. The interpretation of Spearman’s ρ (*r*_*s*_) is similar to that of Pearson’s *r*. Additional explorative sensitivity analyses regarding the prevalence as well categorical and dimensional mean-level and rank-order stability of PD according to specific age ranges at baseline (12–14 and 15–18 years) are presented in the Supplementary Material. All statistical analyses were conducted using RStudio [Version 1.4.1106; (55)]. Statistical significance was set to *p* < 0.05 for all analyses. Complete case analyses were performed.

## Results

### Prevalence Rates of Current Mental Disorders at Baseline and at Follow-Up

Findings regarding the prevalence rates of mental disorders at baseline and at follow-up are presented in Table 1. At baseline, 74 (64.9%) participants reported a current mental-health disorder; conduct disorders (29.8%), anxiety disorders (25.4%), and PDs (20.0%) were the most frequent diagnoses. Fifty-five (61.1%) participants were receiving mental-health treatment at the time of the assessment. At follow-up, the prevalence rate for any mental disorder was about 55.6%; substance-use disorders (35.6%), PDs (30.4%), and ADHD (20.9%) were the most common. A total of 27 (23.5%) participants reported receiving mental-health treatment at follow-up (Table 1). Participants with a PD at follow-up were significantly more likely to report disability insurance than participants without a PD at follow-up [χ^2^(1) = 6.10; *p* = 0.010] (Table 2) [see (56)].

**TABLE 2:** Social benefits at follow-up (t2) (*N* = 115).

	Follow-up (t2)				
	Total sample	No PDs	PDs	χ ^2^	*p*-value
		
	*n* (%)	*n* (%)	*n* (%)		
Social welfare^a^	29 (25.2)	18 (22.5)	11 (31.4)	0.610	0.354
Unemployment insurance^a^	8 (7.0)	5 (6.2)	3 (8.6)	0.003	0.698
Disability insurance^a^	17 (14.8)	7 (8.8)	10 (28.6)	6.102	0.010*

^a^Only available at follow-up. *p < 0.05.

### Prevalence Rates of PDs at Baseline and at Follow-Up

Findings regarding the prevalence rates of PDs at baseline and at follow-up are presented in Table 3. At baseline, 23 (20.0%) participants met the criteria for any PD. While 10 (8.7%) participants met the criteria for one PD diagnosis, 5 (4.3%) met the criteria for two, and 8 (7.0%) met the criteria for three or more PD diagnoses. With a prevalence rate of 8.7%, borderline PD was the most common diagnosis, followed by antisocial PD (6.1%). Every participant with a PD at baseline also met criteria for another type of mental disorder at baseline. At follow-up, the prevalence rate for any PD was 30.4%. Overall, 18 (15.6%) participants met the criteria for only one PD, while 8 (7.0%) had two PD diagnoses, and 9 (7.8%) met the criteria for three or more PD diagnoses. The most frequently diagnosed disorders were antisocial (16.5%), borderline (7.8%), and obsessive-compulsive PDs (7.0%). At the cluster level, cluster B PD disorders were the most prevalent diagnoses, both at baseline (13.9%) and at follow-up (20.0%). All participants with a PD at follow-up, except one, met the criteria for another type of mental disorder.

**TABLE 3:** Prevalence rates of personality disorder diagnoses at baseline (t1) and follow-up (t2) (*N* = 115).

Personality disorders (PDs)	Baseline (t1)	Follow-up (t2)
	
	*n* (%)	*n* (%)
Any PD	23 (20.0)	35 (30.4)
One PD	10 (8.7)	18 (15.6)
Two PDs	5 (4.3)	8 (7.0)
≥Three PDs	8 (7.0)	9 (7.8)
Cluster A	5 (4.3)	8 (7.0)
Paranoid	3 (2.6)	3 (2.6)
Schizotypal	0 (0.0)	2 (1.8)
Schizoid	3 (2.6)	5 (4.3)
Cluster B	16 (13.9)	23 (20.0)
Histrionic	2 (1.7)	0 (0.0)
Narcissistic	4 (3.5)	2 (1.7)
Borderline	10 (8.7)	9 (7.8)
Antisocial^a^	7 (6.1)	19 (16.5)
Cluster C	8 (7.0)	13 (11.3)
Avoidant	3 (2.6)	5 (4.3)
Dependent	1 (0.9)	1 (0.9)
Obsessive compulsive	4 (3.5)	8 (7.0)
PD NOS^b^	3 (2.6)	5 (4.3)
Passive aggressive	5 (4.3)	5 (4.3)
Depressive	4 (3.5)	7 (6.1)

*Participants with multiple PDs are displayed more than once. ^a^Including participants younger than 18 years at baseline. ^b^PD not otherwise specified (NOS).*

### Categorical Stability

Findings regarding the categorical stability of PDs from baseline to follow-up are presented in Table 4.

**TABLE 4:** Categorical stability of personality disorders from baseline (t1) to follow-up (t2) (*N* = 115).

					Mean-level stability	Rank-order stability	
	
Personality disorders (PDs)	Absent t1 and t2	Present t1/absent t2	Absent t1/present t2 (new cases)	Present t1 and t2 (enduring cases)	Proportion enduring^a^	Cohen’s κ	Tetrachoric correlation coefficient

	*n* (%)	*n* (%)	*n* (%)	*n* (%)	%	κ	*r*_*tet*_
Any full-syndrome PD	68 (59.1)	12 (10.4)	24 (20.9)	11 (9.6)	47.8	0.18	0.33***
Cluster A	104 (90.4)	3 (2.6)	6 (5.2)	2 (1.7)	40.0	0.27	0.60***
Paranoid	109 (94.9)	3 (2.6)	3 (2.6)	0 (0.0)	0.0	−0.03	0.38***
Schizotypal	113 (983)	0 (0.0)	2 (1.7)	0 (0.0)	−	−	−
Schizoid	109 (94.8)	1 (0.9)	3 (2.6)	2 (1.7)	66.7	0.48	0.85***
Cluster B	81 (70.4)	11 (9.6)	18 (15.6)	5 (4.3)	31.2	0.11	0.23*
Histrionic	113 (98.3)	2 (1.7)	0 (0.0)	0 (0.0)	0.0	−	−
Narcissistic	109 (94.8)	4 (3.5)	2 (1.7)	0 (0.0)	0.0	−0.02	0.40***
Borderline	97 (84.4)	9 (7.8)	8 (7.0)	1 (0.9)	10.0	0.02	0.08
Antisocial^b^	92 (80.0)	4 (3.5)	16 (13.9)	3 (2.6)	42.9	0.16	0.41***
Cluster C	95 (82.6)	7 (6.0)	12 (10.4)	1 (0.9)	12.5	0.01	0.03
Avoidant	107 (93.0)	3 (2.6)	5 (4.3)	0 (0.0)	0.0	−0.03	0.28**
Dependent	113 (98.3)	1 (0.9)	1 (0.9)	0 (0.0)	0.0	−0.01	0.72***
Obsessive compulsive	104 (90.4)	3 (2.6)	7 (6.0)	1 (0.9)	25.0	0.13	0.38***
PD NOS^c^	107 (93.0)	3 (2.6)	5 (4.3)	0 (0.0)	0.0	−0.03	0.28**
Passive aggressive	105 (91.3)	5 (4.3)	5 (4.3)	0 (0.0)	0.0	−0.04	0.17
Depressive	105 (91.3)	3 (2.6)	6 (5.2)	1 (0.9)	25.0	0.14	0.42***

^a^Calculated by the number of enduring cases divided by the total number of participants meeting a PD at baseline. ^b^Including participants younger than 18 years at baseline. ^c^PD not otherwise specified (NOS). – measures not available, as either baseline or follow-up PD criteria were not met. *p < 0.05, **p < 0.01, ***p < 0.001. The sample size is sufficient to achieve a power ≥0.8 if r_tet_ ≥ 0.42.

#### Mean-Level Stability

The number of enduring cases from baseline to follow-up could only be calculated for PDs diagnosed at baseline. Since no participants met the criteria for a schizotypal PD at baseline, mean-level stability could not be calculated for this disorder. Of the 23 participants who met the criteria for one or more PDs at baseline, 11 still met the criteria for a PD diagnosis at follow-up, resulting in a categorical mean-level stability of 47.8%. Overall, 12 of these 23 participants improved from baseline to follow-up by no longer meeting the criteria for a PD, while 24 of 92 participants with no PD at baseline met the criteria for a PD at follow-up. With only one participant out of 10 meeting the criteria for borderline PD at both assessments, the categorical mean-level stability of borderline PD was low (10.0%). For schizotypal, histrionic, narcissistic, antisocial, avoidant, dependent, PD NOS, and passive-aggressive PDs, none of the participants met the criteria at baseline or at follow-up.

#### Rank-Order Stability

Cohen’s κ and tetrachoric correlations (*r*_*tet*_) could only be calculated for PDs for which there were participants who met the criteria at baseline or at follow-up or at both measurement points. Since no participants met the criteria for a schizotypal PD at baseline, and no participants met the criteria for a histrionic PD at follow-up, Cohen’s κ and tetrachoric correlations (*r*_*tet*_) could not be calculated for either of these disorders. With a Cohen’s κ of 0.18 for any PD, the concordance between baseline and follow-up assessments was low. For individual diagnoses, κ was likewise low, except for schizoid PD (κ = 0.48). The tetrachoric correlation coefficient (*r*_*tet*_) from baseline to follow-up for any PD was 0.33, which indicates a moderate rank-order stability. For individual PDs, rank-order stability ranged from low (borderline, avoidant, PD NOS, and passive-aggressive PDs) to moderate (paranoid, narcissistic, antisocial, obsessive-compulsive, and depressive PDs) to high (schizoid, dependent PDs). With a tetrachoric correlation coefficient (*r*_*tet*_) of 0.60, rank-order stability was by far the highest for cluster A disorders.

### Dimensional Stability

Findings regarding the dimensional stability of PDs from baseline to follow-up are presented in Table 5.

**TABLE 5:** Dimensional stability of personality disorders from baseline to follow-up (*N* = 115).

	Mean-level stability					Rank-order stability
	Baseline	Follow-up				
		
Personality disorder traits	*M* (*SD*)	*M* (*SD*)	Mean difference	Cohen’s *d*	*p-*value	Spearman’s ρ
Total score	99.27 (19.63)	104.1 (18.52)	4.89	0.23	0.016*	0.24**
Cluster A	29.1 (6.94)	31.23 (6.96)	2.13	0.26	0.006**	0.18
Paranoid	9.08 (2.83)	9.90 (2.90)	0.82	0.22	0.017*	0.13
Schizotypal	10.20 (1.93)	10.65 (2.16)	0.44	0.14	0.123	0.11
Schizoid	8.19 (1.92)	9.36 (2.95)	1.17	0.36	<0.001***	0.22*
Cluster B	42.70 (10.11)	43.44 (8.93)	0.74	0.07	0.462	0.28**
Histrionic	9.79 (2.56)	9.20 (1.51)	-0.69	0.24	0.010*	0.28**
Narcissistic	10.82 (2.78)	10.66 (2.41)	-0.15	0.04	0.649	0.23*
Borderline	13.36 (5.05)	12.83 (3.92)	-0.53	0.11	0.236	0.36***
Antisocial	8.73 (2.56)	10.81 (3.70)	2.06	0.57	<0.001***	0.31***
Cluster C	27.47 (5.80)	29.73 (6.34)	2.26	0.30	0.001**	0.20*
Avoidant	9.13 (2.89)	9.18 (2.69)	0.05	0.01	0.864	0.31***
Dependent	9.82 (2.64)	10.14 (2.72)	0.33	0.10	0.289	0.27**
Obsessive compulsive	10.17 (3.05)	11.91 (3.31)	1.75	0.42	<0.001***	−0.08
Passive aggressive	9.17 (3.01)	9.43 (2.64)	0.25	0.06	0.470	0.08
Depressive	9.35 (3.14)	10.41 (3.73)	1.06	0.26	0.005**	0.25**

*p < 0.05, **p < 0.01, ***p < 0.001. The sample size is sufficient to achieve a power of ≥0.8 if d ≥ 0.24 and ρ ≥ 0.23.

#### Mean-Level Stability

Overall, the mean-level scores of dimensional ratings increased for most disorders. The total score significantly increased from baseline to follow-up, although the effect size was small (*d* = 0.23; *p* = 0.016). Significant increases were found for paranoid (*d* = 0.22; *p* = 0.017), schizoid (*d* = 0.36; *p* < 0.001), antisocial (*d* = 0.57; *p* = < 0.001), obsessive-compulsive (*d* = 0.42; *p* < 0.001), and depressive PDs (*d* = 0.26; *p* = 0.005). Findings regarding the mean-level scores for schizotypal, narcissistic, borderline, dependent, and depressive traits revealed no significant change. A significant decrease was found only for histrionic traits, although the effect size was small (*d* = 0.24; *p* = 0.010).

#### Rank-Order Stability

The pattern of rank-order stability of the dimensional scores from baseline to follow-up ranged from low (paranoid, schizoid, schizotypal, histrionic, narcissistic, avoidant, dependent, obsessive-compulsive, passive-aggressive, and depressive) to moderate (borderline, antisocial). Correlations were significant, except for paranoid (*r*_*s*_ = 0.13, p = 0.153), schizotypal (*r*_*s*_ = 0.11, *p* = 0.264), obsessive-compulsive (*r*_*s*_ = −0.08, *p* = 0.412), and passive-aggressive traits (*r*_*s*_ = 0.08, *p* = 0.423).

## Discussion

The aim of the current study was to examine the prevalence rates as well as the mean-level and rank-order stability of PDs over a 10-year follow-up in adolescents placed in residential care and juvenile-justice institutions. Both the stability of PD categories and the stability of dimensional PD traits were analyzed from adolescence to adulthood. The present findings indicated high PD prevalence rates in young adults with a history of child welfare and juvenile-justice placements, while PD diagnoses and PD traits exhibited only low to moderate stability over the 10-year follow-up.

At least three findings have to be discussed in more detail. First, PD prevalence rates substantially increased from adolescence to adulthood in this high-risk sample. While the normative course of BPD during adolescence is described as an increase of BPD pathology from puberty to young adulthood (57), most previous findings indicate a general decline in PDs and PD traits beginning in young adulthood (17). On the other hand, the prevalence rates of any PD as well as of specific PDs are consistent with the existing literature; the prevalence rates of PDs in institutionalized youth and young adults with a history of out-of-home care have been found to range between 18 and 40% across studies (43–45). A recent meta-analysis on mental disorders in incarcerated youth, which included 30 studies of 8,000 participants, indicated that antisocial and borderline PDs were relatively common in both males and females, while the prevalence of narcissistic and schizotypal PDs was comparably low (58). The current study seems to confirm this pattern, as antisocial and borderline PDs were among the most frequently diagnosed disorders, both at baseline and at follow-up. An increase in PD diagnoses from adolescence to adulthood in this sample, may, thus, be explained by the fact that many adolescents in residential care and juvenile-justice institutions have experienced severe childhood adversities (e.g., child abuse and neglect), which are shown to significantly contribute to the development of PDs (59, 60). For instance, the meta-analysis by Porter et al. (37) found that patients with borderline PD were over 13 times more likely to report childhood adversity than non-clinical controls. In addition, participants in this high-risk sample were likely to have experienced a range of other critical risk factors, such as unfavorable parenting practices, low socioeconomic status, childhood psychopathology, including high substance use, self-harming behavior, and youth delinquency, which have also been shown to be significantly associated with the development of PDs over time (38–42). Given the multifaceted nature of problems faced by juveniles in child welfare care and juvenile-justice institutions, the institutions often lack the professional and financial means to detect personality problems at an early stage, leading to delays in diagnoses and appropriate treatment. Delaying appropriate diagnoses, in turn, carries clinical risk, as evidence is accumulating that many of the harms associated with PDs occur early in the course of the disorder (61), and delay tends to lead toward greater impairments and poorer outcomes (62).

Second, on the categorical level, the mean-level stability of any PD was only moderate, and the mean-level stabilities of specific PDs were low to moderate, except for schizoid PD (high). The concordance between baseline and follow-up assessments (i.e., Cohen’s κ) was low, both for any PD and for individual PDs, except for schizoid PD (moderate). The rank-order stability (i.e., tetrachoric correlation (*r*_*tet*_) of any PD category was moderate. For individual diagnoses, the rank-order stability ranged from low (i.e., borderline, avoidant, PD NOS, passive-aggressive PDs) to moderate (i.e., paranoid, narcissistic, antisocial, obsessive-compulsive, depressive PDs) to high (schizoid, dependent PDs). Regarding categorical mean-level stability, Chanen et al. (36) found a higher proportion of enduring cases (74%) compared to our findings (47%), which may be due to the shorter follow-up interval (2 years), the clinical status of participants (outpatients), and the narrower age range (15–18 years old) in their study. Indeed, the explorative age-sensitive analyses in the Supplementary Material revealed a higher categorical mean-level stability for the participants who were 15–18 years old than for the participants who were 12–14 years old, although the stability still seems to be lower than that found by Chanen et al. (36). Categorical mean-level stabilities for individual PDs, however, were similar to those found by Chanen et al. (36). As such, participants may have changed specific PDs (from one PD category to another category) but did not discard the general diagnosis of a PD over time. Noteworthy, however, is that 24 (20.9%) participants first developed a PD in young adulthood. As the explorative age-sensitive analyses revealed, older adolescents (15–18 years) were more likely to meet a PD diagnosis first at follow-up than younger adolescents (12–14 years). This suggests that the onset of a PD indeed lies in later adolescence and that some of the present sample had not yet passed the critical age. Another explanation might be that PDs in (young) adolescence are more difficult to detect (63). In addition, older adolescents with a PD diagnosis between 15 and 18 years may have already had longer and more stable patterns of personality pathology, which, therefore, may be more predictive of unfavorable long-term outcomes. Nevertheless, a total of 12 (10.4%) participants improved from baseline to follow-up and no longer met the criteria for a PD in adulthood. While this could have been due to several factors (e.g., treatment or spontaneous remission), it is also possible that these participants no longer met the diagnosis of a PD but still exhibited PD symptoms. This, in turn, is a major concern of the categorical classification system, as it is based on an arbitrary diagnostic threshold that can be easily met (PD diagnosis) or not met (no PD diagnosis) by an increase or decrease in a single criterion.

Regarding categorical rank-order stability, the poor concordances between the baseline and follow-up assessments (i.e., Cohen’s κ) for any PD and for individual PD diagnoses are consistent with those found by Chanen et al. (36). Findings regarding rank-order stability measured with tetrachoric correlations (*r*_*tet*_) are difficult to compare across studies, since Cohen’s κ remains the most common statistical measure for assessing the rank-order stability of categorical data. Overall, rank-order stability nevertheless seemed to be higher for specific PD diagnoses (i.e., paranoid, narcissistic, avoidant, dependent, PD NOS, and passive-aggressive PDs) than mean-level stability for these PD diagnoses, which suggests that even if the specific diagnoses did not remain the same over time, the rank ordering of participants with such a disorder appeared to be more or less the same. Both the rank-order stability and the mean-level stability of borderline PD were particularly weak, which indicates that on average, neither the category nor the rank ordering of participants with a borderline PD remained the same over time. While this may seem somewhat surprising, it is consistent with the narrative review from Bondurant et al. (64), which suggests that there is only little diagnostic borderline PD stability in adolescence. Interestingly, both Cohen’s κ and tetrachoric correlation coefficients (*r*_*tet*_) were considerably higher for older adolescents at baseline (15–18 years) compared to younger adolescents (12–14 years old) at baseline (see Supplementary Table 2), which suggests that diagnoses in early adolescence should be treated with caution.

Third, on the dimensional level, PD scores significantly increased for most of the disorders, except for schizotypal, avoidant, narcissistic, borderline, dependent, and passive-aggressive traits. Histrionic traits significantly decreased from baseline to follow-up. Effect sizes were generally low, except for antisocial and obsessive-compulsive traits. In contrast to our findings, Johnson et al. (34) found a significant mean-level decline in dimensional ratings from adolescence to adulthood, and Chanen et al. (36) found neither a significant increase nor a decrease in PD traits, except for paranoid (increase), antisocial (increase), and depressive PDs (decrease). One explanation is that the study by Johnson et al. (34) was conducted in a community-based sample, while the study by Chanen et al. (36) was conducted with older adolescent outpatients. The overall low to moderate dimensional rank-order stability in the present study was, however, consistent with the rank-order stability found in the studies by Johnson et al. (34) and Chanen et al. (36). This indicates that although mean-level PD traits tended to increase among adolescents in residential care and juvenile-justice institutions through adulthood, their individual rank ordering seemed to be less stable, emphasizing interindividual differences among participants. The additional explorative age-sensitive analyses revealed higher dimensional mean-level and rank-order stability estimates regarding older participants (15–18 years old) than younger participants (12–14 years old). On the one hand, this highlights the presence of PD traits in early adolescence but on the other hand, suggests that PD diagnoses before the age of 15 should be interpreted with caution.

### Strengths

The current study fills an important gap in the existing literature on the stability of PDs by explicitly presenting findings from adolescence to adulthood in a high-risk sample. Indeed, only a few studies have investigated the stability of PDs from adolescence to adulthood, and to the best of our knowledge, none have yet investigated the stability of PDs from adolescence to adulthood in adolescents in residential care and juvenile-justice institutions. Yet these adolescents have a particularly high risk of developing a PD due to a cumulation of risk factors. Considering the apparent role of developmental tasks in the transition from adolescence to adulthood in the development of PDs, this study is particularly valuable. Another strength of the current study is the long follow-up interval of 10 years. This is noteworthy given that young-adult care leavers (i.e., juveniles who left residential care or juvenile-justice institutions) are often difficult to locate, since many live in rather unstable and changing circumstances (65) or suffer from severe mental-health disorders (66).

### Limitations

Nonetheless, the findings of this study must be interpreted under the consideration of some limitations. First, the relatively small sample size of 115 participants must be emphasized. As a result, the number of cases for categorical PDs were small, which made it difficult to adequately assess categorical stability and, therefore, the results must be interpreted with caution and replications including larger sample sizes are highly needed. Second, although no significant differences were found in the sociodemographic baseline data between included and excluded participants, a selection bias cannot be completely ruled out. Indeed, positive self-selection may occur in longitudinally followed-up high-risk samples, as participants with severe PDs may have declined to participate at follow-up or could not be located due to difficult life circumstances. On the other hand, it may be that participants who remained connected to mental health care were more likely to participate in the current follow-up study, which could explain the high prevalence rates of PDs. Third, the current study only allowed PDs to be assessed using a two-measurement-point design. The amount of change between two measurement points is, however, not fully informative about the shape of each person’s individual growth trajectory. In addition, a two-wave design cannot distinguish true change from measurement error (67) and is unable to evaluate the impact of regression-to-the-mean effects; that is, a statistical artifact making naturally occurring variations look like true changes when particularly large or small scores are followed by scores closer to the mean (68). Fourth, the dimensional approach taken within this study does not precisely correspond to the dimensions within the ICD-11, as the latter go beyond a mere sum of features within a categorical diagnosis. However, the dimensional approach adopted in the current study can be considered as a proxy, as no empirical evidence was yet available for the dimensional approach proposed by the ICD-11 at the time of the baseline study. Finally, while the present study explicitly focused on the stability of PDs from adolescence to adulthood, the cutoff age of 18 years at baseline is somewhat arbitrary, although adulthood is traditionally described as beginning at the age of 18 years. Indeed, based on psychosocial characteristics, recent studies have suggested that emerging adulthood is a period between adolescence (18 years) and full-fledged adulthood (25 years) (69). Specifically, with regard to etiological influences on the development of personality traits, Hopwood et al. (70) defined late adolescence at age 17, emerging adulthood at age 24, and young adulthood at age 29. Future studies should consider the prolongation of adolescence or emerging adulthood, which is currently taking place, especially in Western societies (69), in order to adequately assess the stability of PDs from adolescence to adulthood.

## Conclusion

Three main findings can be drawn from the current study. First, the prevalence rates of PDs in young adults with a history of child welfare and juvenile-justice placements are high. Second, most categorical PD diagnoses and dimensional PD traits increased from adolescence to adulthood in our sample. Third, overall, the findings indicate low to moderate stability of PDs and PD traits from adolescence to adulthood, although the extent of stability differed according to the PD construct (i.e., categorical diagnoses or dimensional traits), the type of stability (i.e., mean-level or rank-order stability) and the specific PD and PD trait being assessed. As a result, the current findings are in accordance with the growing evidence, that PDs are not that stable. This in turn, emphasizes the current shift to a more dimensional model and highlights the use of the upcoming ICD-11 that acknowledges PDs as only “relatively” stable.

## Data Availability Statement

The raw data supporting the conclusions of this article will be made available by the authors, without undue reservation.

## Ethics Statement

The studies involving humans participants were reviewed and approved by the Ethics Committees on Research Involving Humans at the University of Basel and the University of Lausanne (Switzerland) as well as the Institutional Review Board at the University of Ulm (Germany). The follow-up study procedure was approved by the Ethics Committee Northwestern and Central Switzerland. Written informed consent to participate in this study was provided by the participants and the participants’ legal guardian/next of kin, if participants were under 18 years old.

## Author Contributions

Dd’H, MSt, CB, and KS contributed to conceiving and designing the present manuscript. Dd’H wrote the first draft of the manuscript and analyzed the data. Dd’H, DB, SS, and CB collected the data. MSt supervised the data analyses. CB, MB, NJ, MSc, JF, and KS commented on an earlier draft of the article and supervised the entire process. All authors read and approved the final manuscript.

## Conflict of Interest

The authors declare that the research was conducted in the absence of any commercial or financial relationships that could be construed as a potential conflict of interest.

## Publisher’s Note

All claims expressed in this article are solely those of the authors and do not necessarily represent those of their affiliated organizations, or those of the publisher, the editors and the reviewers. Any product that may be evaluated in this article, or claim that may be made by its manufacturer, is not guaranteed or endorsed by the publisher.
