# Supplementary material for: Prevalence and 10-Year Stability of Personality Disorders From Adolescence to Young Adulthood in a High-Risk Sample
Source: Front Psychiatry. 2022 Mar 24;13:840678. doi: 10.3389/fpsyt.2022.840678 (PMC8987201; doi:10.3389/fpsyt.2022.840678)
Supplement: Supplementary file 1 [file Data_Sheet_1.docx]

Supplementary Material

Baseline sample
(*N* = 592)

**Baseline (t0)**

Reason for drop-out (*N* = 81):

- Did not provide informed consent for a possible follow-up (*N* = 81)

Eligible for follow-up
(*N* = 511)

Reason for drop-out (*N* = 280):

- Could not be located (*N* = 8)
- Could not be reached (*N* = 121)
- Refused to participate (*N* = 99)
- Did not provide informed consent (N = 44)
- Were deceased (*N* = 8)

Follow-up sample
(*N* = 231)

**Follow-Up (t2)**

Reason for drop-out (*N* = 116):

- Only online assessment available (*N* = 51)
- No SCID-II baseline assessment (*N* = 42)
- Younger than 12 years at baseline (*N* = 3)
- Older than 18 years at baseline (*N* = 14)
- Missing data on different variables (*N* = 6)

Sample used for analyses

(*N* = 115)

**Figure S1.** Flow-chart of the study sample.

Table S1. Prevalence rates of personality disorder at baseline (t1) according to age groups.

| **Personality disorders (PDs)** | Baseline (t1) | |
| --- | --- | --- |
|  | 12 to 14 years old  (*N* = 40) | 15 to 18 years old  (*N* = 75) |
|  | *n* (%) | *n* (%) |
| Any PD | 5 (12.5) | 18 (24.0) |
| Cluster A | 0 (0.0) | 5 (6.7) |
| Cluster B | 3 (7.5) | 13 (17.3) |
| Cluster C | 2 (5.0) | 6 (8.0) |

*Note.* Due to the small sample sizes, the prevalence rates for PD diagnoses are only

presented on general and cluster level. Participants with multiple PDs are

displayed more than once.

**Table S2.** Categorical stability of personality disorders from baseline (t1) to follow-up (t2) according to age groups.

|  |  |  |  |  | **Mean level stability** | **Rank-order stability** | |
| --- | --- | --- | --- | --- | --- | --- | --- |
| **Personality disorders (PDs)** | Absent t1 & t2 | Present t1 / Absent t2 | Absent t1 / Present t2 (new cases) | Present t1 & t2 (enduring cases) | Proportion Enduring^a^ | Cohen’s κ | Tetrachoric correlation coefficient |
|  |  |  | **12 to 14 years old at baseline (N = 40)** | | |  |  |
|  | *n* (%) | *n* (%) | *n* (%) | *n* (%) | % | κ | *r*_tet_ |
| Any PD | 20 (70.0) | 4 (10.0) | 7 (17.5) | 1 (2.5) ^b^ | 20.0 | 0.00 | 0.0 |
| Cluster A | - | - | - | - | - | - | - |
| Cluster B | 32 (80.0) | 3 (7.5) | 5 (12.5) | 0 (0.0) | 0.0 | 0.00 | 0.02 |
| Cluster C | 36 (90.0) | 2 (5.0) | 2 (5.0) | 0 (0.0) | 0.0 | - 0.05 | 0.37^*^ |
|  |  |  | **15 to 18 years old at baseline (N = 75)** | | |  |  |
|  | *n* (%) | *n* (%) | *n* (%) | *n* (%) | % | κ | *r*_tet_ |
| Any PD | 40 (53.3) | 8 (10.7) | 17 (22.7) | 10 (13.3) | 55.6 | 0.22 | 0.38^***^ |
| Cluster A | 65 (86.7) | 3 (4.0) | 5 (6.7) | 2 (2.7) | 40.0 | 0.28 | 0.57^***^ |
| Cluster B | 49 (65.3) | 8 (10.7) | 13 (17.3) | 5 (6.7) | 38.5 | 0.15 | 0.29^*^ |
| Cluster C | 59 (78.7) | 5 (6.7) | 10 (13.3) | 1 (1.3) | 16.7 | 0.02 | 0.05 |

*Note.* ^a^ Calculated by the number of enduring cases divided by the total number of participants meeting a PD at baseline. ^b^ This case consists in a PD NOS, therefore it is not displayed on cluster level.

- measures not available, as either baseline or follow-up PD criteria were not met. ^*^p < 0.05, ^**^p < 0.01, ^***^p < 0.001. The sample size is sufficient to achieve a power ≥ 0.8, if r_tet_ ≥ 0.39.

**Table S3.** Dimensional stability of personality disorders from baseline to follow-up according to age groups.

|  | **Mean-level stability** | | | | | **Rank-order stability** |
| --- | --- | --- | --- | --- | --- | --- |
|  |  |  | **12 to 14 years old at baseline (*N* = 40)** | | | |
|  | Baseline | Follow-up |  | | | |
| Personality disorder traits | *M* (*SD*) | *M* (*SD*) | Mean difference | Cohens’ *d* | *p* value | Spearman’s ρ |
| Total score | 97.7 (18.42) | 100.2 (16.13) | 2.5 | 0.12 | 0.462 | 0.06 |
| Cluster A | 29.77 (7.71) | 30.3 (6.079) | 0.52 | 0.07 | 0.664 | 0.28 |
| Cluster B | 40.45 (8.74) | 41.5 (7.93) | 1.05 | 0.10 | 0.535 | 0.07 |
| Cluster C | 27.48 (5.17) | 28.4 (4.8) | 0.92 | 0.14 | 0.376 | 0.00 |
|  | **15 to 18 years old at baseline (*N* = 75)** | | | | | |
|  | Baseline | Follow-up |  |  |  |  |
|  | *M* (*SD*) | *M* (*SD*) | Mean difference | Cohens’ *d* | *p* value | Spearman’s ρ |
| Total score | 100.1 (20.4) | 106.2 (19.59) | 6.20 | 0.29 | 0.016 | 0.30^**^ |
| Cluster A | 28.75 (6.55) | 31.73 (7.42) | 2.99 | 0.35 | 0.003 | 0.14 |
| Cluster B | 43.89 (10.65) | 44.49 (9.31) | 0.57 | 0.05 | 0.652 | 0.33^**^ |
| Cluster C | 27.47 (6.14) | 30.45 (7.04) | 2.99 | 0.38 | 0.001 | 0.25^*^ |

*Note.* ^*^p < 0.05, ^**^p < 0.01, ^***^ p < 0.001. The sample size is sufficient to achieve a power ≥ 0.8, if d ≥ 0.24 and ρ ≥ 0.23.
